# Supplementary material for: Evaluation of Tunisian wheat endophytes as plant growth promoting bacteria and biological control agents against Fusarium culmorum
Source: PLoS One. 2024 May 17;19(5):e0300791. doi: 10.1371/journal.pone.0300791 (PMC11101125; doi:10.1371/journal.pone.0300791)
Supplement: S1 Fig — (PDF) [file pone.0300791.s001.pdf]

S1 Figure

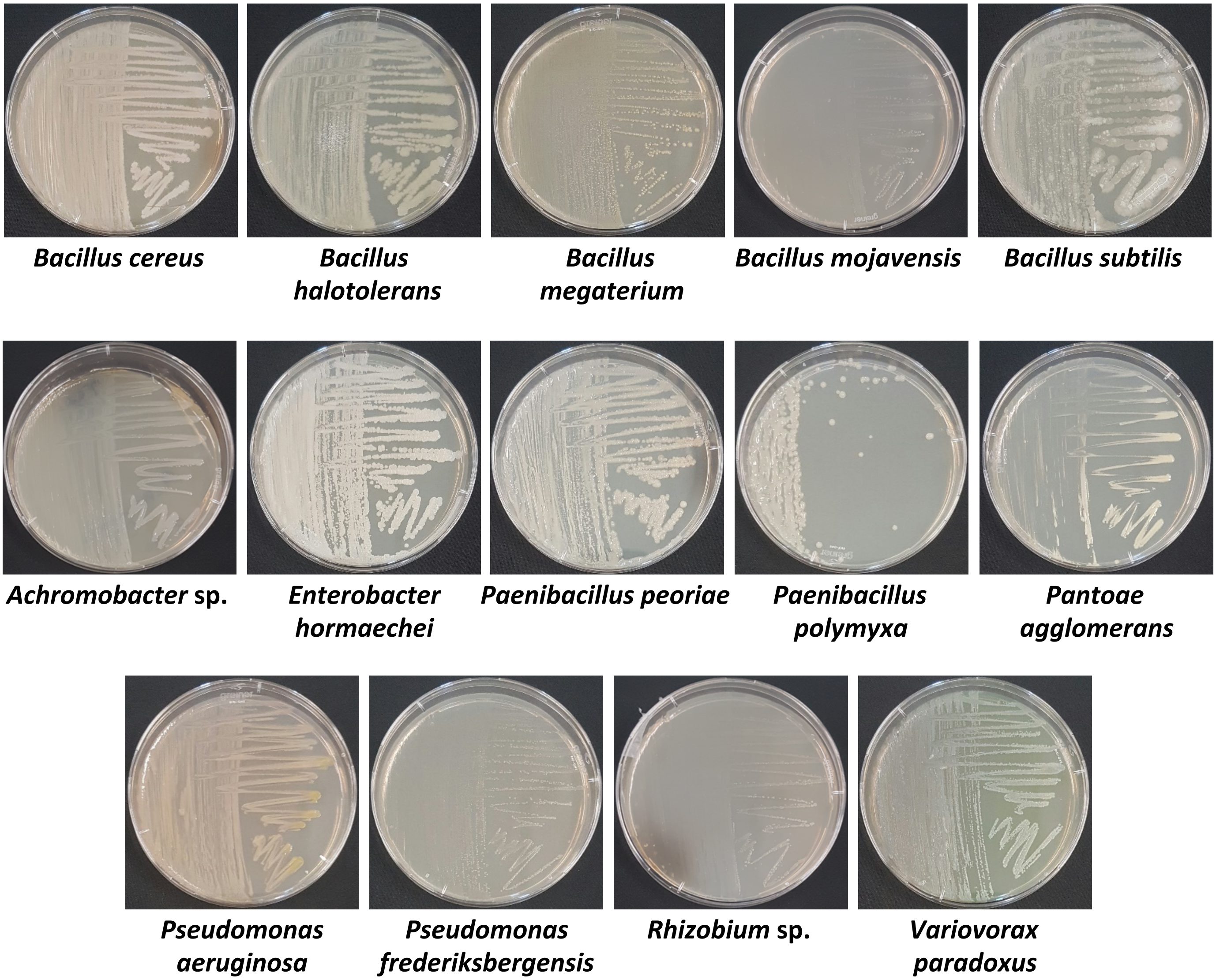

S1 Figure. Macroscopic aspect of the 14 isolated bacterial endophytes growing on agar-solidified lysogeny broth (LB) medium after 48 h of incubation at 37°C.
