# Supplementary material for: Evaluation of Tunisian wheat endophytes as plant growth promoting bacteria and biological control agents against Fusarium culmorum
Source: PLoS One. 2024 May 17;19(5):e0300791. doi: 10.1371/journal.pone.0300791 (PMC11101125; doi:10.1371/journal.pone.0300791)
Supplement: S2 Table — (PDF) [file pone.0300791.s005.pdf]

S2 Table. Statistical analysis of the transcriptomic data.

iTPS	Feuille Log2(ddCt)	Feuille SE	Feuille t-st.	Racine Log2(ddCt)	Racine SE	Racine t-st.
Control	0,00	0,09	/	0,00	0,08	/
Achromobacter sp.	2,91	0,14	0,0009	1,20	0,14	0,0250
Bacillus cereus	2,94	0,33	0,0010	0,88	0,20	0,0460
Bacillus halotolerans	2,29	0,10	0,0050	0,90	0,24	0,0490
Bacillus megaterium	1,15	0,38	0,0540	1,03	0,36	0,0240
Bacillus mojavensis	2,83	0,13	0,0009	1,31	0,12	0,0120
Bacillus subtilis	0,86	0,07	0,0190	1,22	0,22	0,0230
Enterobacter hormaechei	2,28	0,19	0,0020	0,58	0,19	0,0950
Paenibacillus peoriae	1,51	0,20	0,0030	0,97	0,16	0,0330
Paenibacillus polymyxa	1,11	0,36	0,0330	0,22	0,11	0,5870
Pantoae agglomerans	1,53	0,12	0,0050	1,31	0,16	0,0140
Pseudomonas aeruginosa	0,89	0,10	0,0140	1,60	0,46	0,0910
Pseudo frederiksbergensis	2,08	0,12	0,0050	1,01	0,29	0,0260
Rhizobium sp.	0,86	0,11	0,0140	1,29	0,13	0,0110
Variovorax paradoxus	2,74	0,26	0,0007	1,38	0,25	0,0090

CKX	Feuille Log2(ddCt)	Feuille SE	Feuille t-st.	Racine Log2(ddCt)	Racine SE	Racine t-st.
Control	0,00	0,03	/	0,00	0,03	/
Achromobacter sp.	-1,80	0,30	0,0120	-2,15	0,24	0,0080
Bacillus cereus	-2,45	0,41	0,0150	-1,49	0,20	0,0130
Bacillus halotolerans	-0,58	0,40	0,2650	-0,34	0,16	0,3440
Bacillus megaterium	-1,12	0,10	0,0020	-0,93	0,45	0,0230
Bacillus mojavensis	-0,29	0,19	0,2350	-0,52	0,32	0,0720
Bacillus subtilis	-0,74	0,33	0,1510	-0,72	0,30	0,0350
Enterobacter hormaechei	-0,97	0,11	0,0040	-0,61	0,38	0,0520
Paenibacillus peoriae	-0,53	0,17	0,0700	-1,50	0,06	0,0090
Paenibacillus polymyxa	-0,92	0,18	0,0200	-0,19	0,17	0,4770
Pantoae agglomerans	-0,70	0,06	0,0090	-0,15	0,42	0,6760
Pseudomonas aeruginosa	-0,86	0,12	0,0300	-0,57	0,11	0,0630
Pseudo frederiksbergensis	-0,82	0,08	0,0050	-0,12	0,32	0,4370
Rhizobium sp.	-1,07	0,18	0,0150	-3,24	0,53	0,0010
Variovorax paradoxus	-1,46	0,05	0,0020	-0,90	0,26	0,0350

WAK6	Feuille Log2(ddCt)	Feuille SE	Feuille t-st.	Racine Log2(ddCt)	Racine SE	Racine t-st.
Control	0,00	0,11	/	0,00	0,21	/
Achromobacter sp.	1,51	0,13	0,0050	-2,00	0,54	0,0290
Bacillus cereus	1,83	0,32	0,0360	-1,66	0,59	0,0280
Bacillus halotolerans	2,51	0,26	0,0090	-1,03	0,35	0,0820
Bacillus megaterium	1,56	0,11	0,0030	-0,61	0,29	0,1630
Bacillus mojavensis	1,86	0,18	0,0060	-1,39	0,05	0,0680
Bacillus subtilis	1,88	0,12	0,0006	-1,57	0,17	0,0770
Enterobacter hormaechei	1,44	0,17	0,0160	-2,34	0,54	0,0080
Paenibacillus peoriae	1,75	0,13	0,0020	-1,86	0,49	0,0280
Paenibacillus polymyxa	2,12	0,38	0,0380	-3,39	0,53	0,0020
Pantoae agglomerans	2,14	0,68	0,1110	-1,06	0,48	0,0660
Pseudomonas aeruginosa	1,34	0,13	0,0220	-2,37	0,54	0,0580
Pseudo frederiksbergensis	-0,23	0,15	0,5720	-0,92	0,30	0,1130
Rhizobium sp.	1,79	0,17	0,0080	-0,14	0,35	0,4100
Variovorax paradoxus	0,79	0,10	0,0110	-0,37	0,47	0,2520

LRR-RLK	Feuille Log2(ddCt)	Feuille SE	Feuille t-st.	Racine Log2(ddCt)	Racine SE	Racine t-st.
Control	0,00	0,23	/	0,00	0,08	/
Achromobacter sp.	2,69	0,19	0,0030	1,80	0,51	0,0050
Bacillus cereus	1,35	0,33	0,0910	0,95	0,39	0,0480
Bacillus halotolerans	3,08	0,47	0,0310	2,15	0,48	0,0030
Bacillus megaterium	1,26	0,21	0,0400	1,94	0,41	0,0030
Bacillus mojavensis	1,56	0,29	0,0550	0,80	0,23	0,0440
Bacillus subtilis	1,10	0,09	0,0030	1,97	0,36	0,0020
Enterobacter hormaechei	2,03	0,24	0,0190	0,74	0,56	0,0420
Paenibacillus peoriae	3,13	0,19	0,0030	2,63	0,37	0,0030
Paenibacillus polymyxa	1,35	0,14	0,0060	1,44	0,16	0,0150
Pantoae agglomerans	2,62	0,50	0,0510	3,34	0,39	0,0290
Pseudomonas aeruginosa	0,82	0,07	0,0060	1,19	0,20	0,0290
Pseudo frederiksbergensis	1,27	0,08	0,0008	1,00	0,09	0,0570
Rhizobium sp.	0,41	0,25	0,2030	1,35	0,17	0,0600
Variovorax paradoxus	3,25	0,44	0,0250	3,01	0,50	0,0050

EDS1	Feuille Log2(ddCt)	Feuille SE	Feuille t-st.	Racine Log2(ddCt)	Racine SE	Racine t-st.
Control	0,00	0,23	/	0,00	0,22	/
Achromobacter sp.	0,88	0,12	0,0190	0,63	0,17	0,0510
Bacillus cereus	0,70	0,16	0,0590	0,73	0,07	0,0380
Bacillus halotolerans	2,16	0,36	0,0120	1,61	0,18	0,0030
Bacillus megaterium	0,88	0,13	0,0200	0,77	0,24	0,0290
Bacillus mojavensis	1,33	0,10	0,0040	0,39	0,18	0,3090
Bacillus subtilis	0,92	0,10	0,0120	0,34	0,21	0,2140
Enterobacter hormaechei	0,73	0,14	0,0410	1,44	0,27	0,0040
Paenibacillus peoriae	1,08	0,02	0,0180	-0,07	0,14	0,8890
Paenibacillus polymyxa	0,79	0,07	0,0230	0,68	0,09	0,0890
Pantoae agglomerans	0,55	0,27	0,2630	0,50	0,24	0,0800
Pseudomonas aeruginosa	1,55	0,23	0,0190	1,25	0,46	0,0760
Pseudo frederiksbergensis	1,16	0,14	0,0060	0,97	0,21	0,0200
Rhizobium sp.	1,37	0,35	0,0400	0,29	0,09	0,3520
Variovorax paradoxus	0,44	0,15	0,2070	0,54	0,25	0,0770

MAPK3	Feuille Log2(ddCt)	Feuille SE	Feuille t-st.	Racine Log2(ddCt)	Racine SE	Racine t-st.
Control	0,00	0,08	/	0,00	0,07	/
Achromobacter sp.	0,12	0,09	0,0220	0,83	0,21	0,0200
Bacillus cereus	0,72	0,14	0,0100	0,66	0,16	0,0580
Bacillus halotolerans	1,37	0,34	0,0370	1,24	0,23	0,0020
Bacillus megaterium	0,72	0,11	0,0020	1,11	0,29	0,0030
Bacillus mojavensis	1,01	0,15	0,0060	0,83	0,16	0,0350
Bacillus subtilis	0,55	0,08	0,0020	0,59	0,31	0,0170
Enterobacter hormaechei	1,10	0,19	0,0140	0,22	0,28	0,1040
Paenibacillus peoriae	0,09	0,07	0,0220	0,15	0,24	0,2370
Paenibacillus polymyxa	0,27	0,06	0,0070	0,41	0,07	0,2670
Pantoae agglomerans	1,00	0,13	0,0040	1,14	0,21	0,0280
Pseudomonas aeruginosa	0,91	0,13	0,0004	0,68	0,14	0,0090
Pseudo frederiksbergensis	0,35	0,07	0,0040	0,81	0,11	0,1230
Rhizobium sp.	0,32	0,13	0,0180	0,07	0,21	0,2900
Variovorax paradoxus	0,20	0,02	0,0170	0,96	0,11	0,0360

NPR1	Feuille Log2(ddCt)	Feuille SE	Feuille t-st.	Racine Log2(ddCt)	Racine SE	Racine t-st.
Control	0,00	0,15	/	0,00	0,26	/
Achromobacter sp.	0,71	0,17	0,0430	1,75	0,23	0,0004
Bacillus cereus	1,28	0,31	0,0130	1,55	0,27	0,0008
Bacillus halotolerans	1,24	0,07	0,0260	1,93	0,37	0,0004
Bacillus megaterium	0,84	0,04	0,0400	1,89	0,21	0,0004
Bacillus mojavensis	1,60	0,30	0,0090	1,52	0,16	0,0020
Bacillus subtilis	1,47	0,17	0,0140	1,47	0,27	0,0120
Enterobacter hormaechei	1,00	0,20	0,0200	1,82	0,29	0,0120
Paenibacillus peoriae	0,19	0,16	0,2240	-0,47	0,29	0,1030
Paenibacillus polymyxa	0,36	0,06	0,1240	-0,48	0,15	0,1090
Pantoae agglomerans	1,33	0,18	0,0160	1,39	0,45	0,0300
Pseudomonas aeruginosa	1,24	0,05	0,0250	1,31	0,21	0,0120
Pseudo frederiksbergensis	1,38	0,06	0,0230	1,92	0,23	0,0010
Rhizobium sp.	1,38	0,18	0,0170	1,48	0,26	0,0120
Variovorax paradoxus	1,32	0,37	0,0130	1,88	0,24	0,0003

PR1	Feuille Log2(ddCt)	Feuille SE	Feuille t-st.	Racine Log2(ddCt)	Racine SE	Racine t-st.
Control	0,00	0,30	/	0,00	0,15	/
Achromobacter sp.	1,71	0,38	0,0410	-0,02	0,48	0,9520
Bacillus cereus	1,25	0,14	0,0050	0,04	0,19	0,8810
Bacillus halotolerans	2,82	0,34	0,0080	0,67	0,17	0,2170
Bacillus megaterium	0,40	0,09	0,0950	0,67	0,30	0,4220
Bacillus mojavensis	1,34	0,14	0,0040	1,10	0,18	0,1240
Bacillus subtilis	1,07	0,24	0,0220	-0,22	0,42	0,7120
Enterobacter hormaechei	2,70	0,37	0,0130	0,78	0,27	0,3690
Paenibacillus peoriae	0,28	0,39	0,4640	-0,77	0,27	0,1150
Paenibacillus polymyxa	0,47	0,27	0,1830	-0,99	0,19	0,0580
Pantoae agglomerans	1,37	0,17	0,0050	1,74	0,12	0,0240
Pseudomonas aeruginosa	0,28	0,22	0,1990	-1,04	0,05	0,0470
Pseudo frederiksbergensis	0,43	0,11	0,0810	-1,74	0,16	0,0100
Rhizobium sp.	1,16	0,12	0,0060	2,37	0,17	0,0240
Variovorax paradoxus	0,38	0,18	0,1480	1,74	0,26	0,0300

CHT2	Feuille Log2(ddCt)	Feuille SE	Feuille t-st.	Racine Log2(ddCt)	Racine SE	Racine t-st.
Control	0,00	0,11	/	0,00	0,28	/
Achromobacter sp.	-0,98	0,24	0,0380	-0,69	0,09	0,0150
Bacillus cereus	0,74	0,10	0,0240	0,41	0,08	0,1900
Bacillus halotolerans	1,93	0,26	0,0070	0,94	0,22	0,0510
Bacillus megaterium	0,40	0,15	0,1390	0,44	0,09	0,2580
Bacillus mojavensis	0,74	0,05	0,0290	1,18	0,10	0,0030
Bacillus subtilis	0,84	0,16	0,0240	0,30	0,10	0,4350
Enterobacter hormaechei	1,07	0,26	0,0460	0,31	0,18	0,2030
Paenibacillus peoriae	0,79	0,19	0,0350	0,38	0,12	0,1030
Paenibacillus polymyxa	0,43	0,13	0,1120	0,53	0,11	0,0500
Pantoae agglomerans	0,39	0,15	0,1680	0,54	0,12	0,0630
Pseudomonas aeruginosa	0,70	0,12	0,0290	1,07	0,04	0,0100
Pseudo frederiksbergensis	0,56	0,23	0,1160	0,63	0,13	0,1480
Rhizobium sp.	0,95	0,21	0,0300	-0,53	0,05	0,0470
Variovorax paradoxus	0,22	0,12	0,3470	0,20	0,22	0,4060

PR5	Feuille Log2(ddCt)	Feuille SE	Feuille t-st.	Racine Log2(ddCt)	Racine SE	Racine t-st.
Control	0,00	0,22	/	0,00	0,32	/
Achromobacter sp.	2,16	0,38	0,0050	1,91	0,45	0,0080
Bacillus cereus	2,15	0,54	0,0190	0,89	0,03	0,0230
Bacillus halotolerans	0,37	0,13	0,2410	0,31	0,30	0,4000
Bacillus megaterium	0,22	0,38	0,7960	0,27	0,36	0,5560
Bacillus mojavensis	1,33	0,25	0,0190	1,75	0,22	0,0170
Bacillus subtilis	0,10	0,11	0,7770	0,56	0,22	0,2920
Enterobacter hormaechei	3,37	0,41	0,0020	1,99	0,44	0,0010
Paenibacillus peoriae	1,87	0,30	0,0060	1,78	0,11	0,0050
Paenibacillus polymyxa	1,55	0,29	0,0140	1,90	0,49	0,0460
Pantoae agglomerans	0,72	0,45	0,3340	0,52	0,30	0,1900
Pseudomonas aeruginosa	1,90	0,29	0,0060	2,55	0,52	0,0050
Pseudo frederiksbergensis	1,85	0,46	0,0260	1,26	0,37	0,0060
Rhizobium sp.	-0,63	0,07	0,0210	-1,06	0,26	0,1540
Variovorax paradoxus	3,06	0,25	0,0040	1,25	0,20	0,0080

POD	Feuille Log2(ddCt)	Feuille SE	Feuille t-st.	Racine Log2(ddCt)	Racine SE	Racine t-st.
Control	0,00	0,06	/	0,00	0,16	/
Achromobacter sp.	0,20	0,13	0,0720	-0,13	0,09	0,6180
Bacillus cereus	1,86	0,48	0,0250	1,47	0,23	0,0230
Bacillus halotolerans	2,62	0,03	0,0020	0,14	0,01	0,4910
Bacillus megaterium	-0,03	0,15	0,2310	0,59	0,14	0,0320
Bacillus mojavensis	2,03	0,33	0,0050	1,07	0,31	0,0620
Bacillus subtilis	0,83	0,16	0,0080	1,18	0,12	0,0150
Enterobacter hormaechei	0,06	0,18	0,1670	-0,09	0,07	0,6000
Paenibacillus peoriae	1,82	0,15	0,0009	0,12	0,05	0,5420
Paenibacillus polymyxa	0,90	0,15	0,0050	1,35	0,13	0,0950
Pantoae agglomerans	1,43	0,42	0,0280	0,52	0,08	0,1350
Pseudomonas aeruginosa	2,35	0,33	0,0030	1,08	0,11	0,0110
Pseudo frederiksbergensis	0,38	0,22	0,0660	0,05	0,16	0,8180
Rhizobium sp.	-1,15	0,31	0,1080	-2,43	0,32	0,0270
Variovorax paradoxus	-0,07	0,06	0,2390	-0,24	0,23	0,1630

DEF	Feuille Log2(ddCt)	Feuille SE	Feuille t-st.	Racine Log2(ddCt)	Racine SE	Racine t-st.
Control	0,00	0,17	/	0,00	0,09	/
Achromobacter sp.	-0,27	0,14	0,1760	-0,99	0,10	0,0050
Bacillus cereus	-0,58	0,01	0,0390	-1,12	0,18	0,0200
Bacillus halotolerans	-0,49	0,19	0,0970	-0,81	0,28	0,1100
Bacillus megaterium	-0,41	0,06	0,0560	-0,41	0,07	0,2570
Bacillus mojavensis	-1,00	0,27	0,0490	-1,38	0,23	0,0040
Bacillus subtilis	-0,81	0,12	0,0100	-0,32	0,09	0,4910
Enterobacter hormaechei	0,16	0,19	0,5620	0,04	0,20	0,8200
Paenibacillus peoriae	-1,65	0,29	0,0100	-1,76	0,24	0,0010
Paenibacillus polymyxa	-1,05	0,32	0,0570	-0,24	0,04	0,5700
Pantoae agglomerans	0,80	0,27	0,1010	0,34	0,06	0,4530
Pseudomonas aeruginosa	-0,94	0,08	0,0060	-1,65	0,25	0,0040
Pseudo frederiksbergensis	-0,90	0,21	0,0310	-0,15	0,15	0,8100
Rhizobium sp.	-0,94	0,03	0,0060	0,83	0,16	0,0350
Variovorax paradoxus	-0,44	0,14	0,0680	0,20	0,21	0,2950

PR17	Feuille Log2(ddCt)	Feuille SE	Feuille t-st.	Racine Log2(ddCt)	Racine SE	Racine t-st.
Control	0,00	0,07	/	0,00	0,04	/
Achromobacter sp.	-2,43	0,21	0,0030	-2,19	0,30	0,0020
Bacillus cereus	-1,06	0,43	0,1350	-1,14	0,35	0,0120
Bacillus halotolerans	-0,76	0,23	0,0770	-1,66	0,30	0,0640
Bacillus megaterium	-1,83	0,47	0,0520	-1,00	0,11	0,1150
Bacillus mojavensis	-0,56	0,26	0,2010	-1,59	0,49	0,0030
Bacillus subtilis	-0,74	0,44	0,2730	-1,63	0,06	0,0040
Enterobacter hormaechei	-0,75	0,27	0,1310	-1,92	0,53	0,0360
Paenibacillus peoriae	-2,07	0,06	0,0010	-2,09	0,24	0,0020
Paenibacillus polymyxa	-2,99	0,22	0,0050	-1,37	0,15	0,0590
Pantoae agglomerans	0,16	0,28	0,5390	-0,28	0,27	0,4770
Pseudomonas aeruginosa	-0,72	0,31	0,1630	-1,16	0,07	0,0120
Pseudo frederiksbergensis	-1,35	0,08	0,0020	-1,18	0,25	0,0100
Rhizobium sp.	-2,20	0,66	0,1060	-2,35	0,56	0,0010
Variovorax paradoxus	-0,50	0,14	0,0940	-1,95	0,44	0,0020

THIONIN	Feuille Log2(ddCt)	Feuille SE	Feuille t-st.	Racine Log2(ddCt)	Racine SE	Racine t-st.
Control	0,00	0,10	/	0,00	0,11	/
Achromobacter sp.	3,85	0,89	0,0080	-6,07	0,02	0,0002
Bacillus cereus	3,28	0,74	0,0060	-4,93	0,93	0,0180
Bacillus halotolerans	3,31	0,41	0,0004	-3,42	0,22	0,0002
Bacillus megaterium	2,10	0,17	0,0009	-3,96	0,62	0,0020
Bacillus mojavensis	1,13	0,21	0,0100	-2,47	0,82	0,0200
Bacillus subtilis	1,59	0,07	0,0050	-2,53	0,33	0,0030
Enterobacter hormaechei	3,27	0,42	0,0004	-4,58	0,53	0,0001
Paenibacillus peoriae	0,84	0,26	0,0340	-3,12	0,16	0,0008
Paenibacillus polymyxa	1,16	0,15	0,0040	-2,35	0,21	0,0110
Pantoae agglomerans	2,02	0,54	0,0140	-2,78	0,19	0,0090
Pseudomonas aeruginosa	2,33	0,26	0,0007	-3,31	0,10	0,0004
Pseudo frederiksbergensis	1,92	0,53	0,0160	-3,87	0,44	0,0010
Rhizobium sp.	0,28	0,04	0,1810	-0,43	0,10	0,3020
Variovorax paradoxus	2,97	0,36	0,0004	-4,73	0,41	0,0008

GRP	Feuille Log2(ddCt)	Feuille SE	Feuille t-st.	Racine Log2(ddCt)	Racine SE	Racine t-st.
Control	0,00	0,16	/	0,00	0,25	/
Achromobacter sp.	0,76	0,10	0,0380	-0,34	0,04	0,1470
Bacillus cereus	0,87	0,17	0,0350	-0,37	0,15	0,2980
Bacillus halotolerans	1,74	0,27	0,0240	-0,43	0,15	0,2770
Bacillus megaterium	1,45	0,26	0,0110	-0,50	0,09	0,0180
Bacillus mojavensis	1,67	0,28	0,0100	-0,94	0,09	0,0050
Bacillus subtilis	1,35	0,28	0,0160	-0,78	0,12	0,0060
Enterobacter hormaechei	1,09	0,16	0,0170	-1,02	0,10	0,0150
Paenibacillus peoriae	0,79	0,05	0,0510	-0,95	0,03	0,0040
Paenibacillus polymyxa	1,01	0,21	0,0320	-0,52	0,04	0,0180
Pantoae agglomerans	1,20	0,12	0,0060	-0,67	0,09	0,0100
Pseudomonas aeruginosa	1,58	0,21	0,0030	-1,13	0,15	0,0020
Pseudo frederiksbergensis	1,07	0,07	0,0120	-0,87	0,20	0,0100
Rhizobium sp.	-0,81	0,10	0,0150	-1,35	0,05	0,0020
Variovorax paradoxus	0,57	0,18	0,0830	-0,58	0,13	0,1550

LTP	Feuille Log2(ddCt)	Feuille SE	Feuille t-st.	Racine Log2(ddCt)	Racine SE	Racine t-st.
Control	0,00	0,24	/	0,00	0,15	/
Achromobacter sp.	1,62	0,28	0,0340	2,42	0,25	0,0020
Bacillus cereus	3,09	0,57	0,0080	1,52	0,08	0,0030
Bacillus halotolerans	2,04	0,34	0,0190	2,20	0,25	0,0020
Bacillus megaterium	1,29	0,24	0,0520	1,26	0,27	0,0330
Bacillus mojavensis	0,74	0,12	0,1570	1,14	0,09	0,0110
Bacillus subtilis	0,99	0,12	0,1070	1,13	0,10	0,0080
Enterobacter hormaechei	-1,09	0,22	0,0360	-0,69	0,31	0,0490
Paenibacillus peoriae	-0,72	0,17	0,0930	-0,57	0,15	0,0720
Paenibacillus polymyxa	-1,21	0,14	0,0130	-0,89	0,08	0,0460
Pantoae agglomerans	1,16	0,06	0,0940	2,58	0,15	0,0010
Pseudomonas aeruginosa	1,05	0,11	0,0900	0,88	0,06	0,0270
Pseudo frederiksbergensis	1,78	0,10	0,0390	2,29	0,42	0,0010
Rhizobium sp.	1,85	0,15	0,0360	2,19	0,06	0,0010
Variovorax paradoxus	1,32	0,14	0,0600	1,29	0,15	0,0140

PAL	Feuille Log2(ddCt)	Feuille SE	Feuille t-st.	Racine Log2(ddCt)	Racine SE	Racine t-st.
Control	0,00	0,20	/	0,00	0,11	/
Achromobacter sp.	0,26	0,11	0,2480	1,27	0,25	0,0010
Bacillus cereus	0,77	0,37	0,1380	0,55	0,09	0,2260
Bacillus halotolerans	0,87	0,71	0,3450	0,73	0,10	0,1560
Bacillus megaterium	0,36	0,15	0,1670	1,05	0,33	0,0040
Bacillus mojavensis	1,50	0,16	0,0020	1,00	0,33	0,2690
Bacillus subtilis	0,66	0,17	0,0430	0,87	0,10	0,0060
Enterobacter hormaechei	0,51	0,03	0,0680	0,96	0,13	0,1030
Paenibacillus peoriae	2,43	0,21	0,0020	0,71	0,09	0,1810
Paenibacillus polymyxa	0,92	0,22	0,0260	1,33	0,36	0,0060
Pantoae agglomerans	1,69	0,33	0,0160	1,46	0,29	0,0190
Pseudomonas aeruginosa	1,61	0,27	0,0040	1,16	0,36	0,0920
Pseudo frederiksbergensis	0,68	0,11	0,0250	0,71	0,18	0,0240
Rhizobium sp.	1,07	0,04	0,0140	-4,21	0,37	0,0005
Variovorax paradoxus	-0,35	0,32	0,4210	-0,64	0,04	0,0660

PPO	Feuille Log2(ddCt)	Feuille SE	Feuille t-st.	Racine Log2(ddCt)	Racine SE	Racine t-st.
Control	0,00	0,17	/	0,00	0,28	/
Achromobacter sp.	-0,71	0,09	0,0200	-1,83	0,09	0,0006
Bacillus cereus	-0,57	0,37	0,3160	-2,18	0,16	0,0003
Bacillus halotolerans	-0,45	0,24	0,2780	-1,38	0,22	0,0030
Bacillus megaterium	-2,44	0,21	0,0080	-1,53	0,29	0,0330
Bacillus mojavensis	-1,37	0,21	0,0190	-1,86	0,44	0,0590
Bacillus subtilis	-1,68	0,06	0,0007	-2,13	0,20	0,0010
Enterobacter hormaechei	-0,79	0,12	0,0160	-2,28	0,28	0,0050
Paenibacillus peoriae	-2,34	0,12	0,0007	-1,69	0,22	0,0110
Paenibacillus polymyxa	-1,64	0,08	0,0007	-1,75	0,38	0,0560
Pantoae agglomerans	-0,56	0,12	0,0640	-0,90	0,19	0,0960
Pseudomonas aeruginosa	-1,57	0,17	0,0370	-2,33	0,21	0,0100
Pseudo frederiksbergensis	-2,05	0,24	0,0200	-2,20	0,20	0,0002
Rhizobium sp.	-2,19	0,25	0,0170	-0,67	0,14	0,0510
Variovorax paradoxus	-1,18	0,15	0,0140	-1,94	0,38	0,0590

SQTS	Feuille Log2(ddCt)	Feuille SE	Feuille t-st.	Racine Log2(ddCt)	Racine SE	Racine t-st.
Control	0,00	0,11	/	0,00	0,15	/
Achromobacter sp.	4,34	0,16	0,0002	2,67	0,14	0,0060
Bacillus cereus	3,71	0,82	0,0160	-0,37	0,74	0,3340
Bacillus halotolerans	4,79	0,36	0,0002	-0,36	0,47	0,6180
Bacillus megaterium	1,48	0,27	0,0090	-0,29	0,70	0,3990
Bacillus mojavensis	1,50	0,39	0,0320	-0,43	0,23	0,6240
Bacillus subtilis	2,25	0,16	0,0005	0,20	1,01	0,8570
Enterobacter hormaechei	4,69	0,24	0,0001	2,34	0,56	0,0080
Paenibacillus peoriae	-1,13	0,12	0,0040	-1,34	0,15	0,0580
Paenibacillus polymyxa	-0,96	0,23	0,0810	-1,68	0,35	0,0230
Pantoae agglomerans	3,32	0,15	0,0003	0,92	0,85	0,5250
Pseudomonas aeruginosa	1,56	0,34	0,0090	-1,24	0,28	0,0650
Pseudo frederiksbergensis	2,50	0,65	0,0310	-1,73	0,14	0,0430
Rhizobium sp.	1,67	0,39	0,0160	4,28	0,35	0,0040
Variovorax paradoxus	3,94	0,26	0,0001	3,05	0,46	0,0090

AQP	Feuille Log2(ddCt)	Feuille SE	Feuille t-st.	Racine Log2(ddCt)	Racine SE	Racine t-st.
Control	0,00	0,19	/	0,00	0,13	/
Achromobacter sp.	0,94	0,06	0,0050	0,95	0,35	0,018
Bacillus cereus	0,82	0,06	0,0100	1,05	0,33	0,027
Bacillus halotolerans	1,91	0,37	0,0040	0,59	0,08	0,196
Bacillus megaterium	0,39	0,14	0,3740	1,17	0,28	0,008
Bacillus mojavensis	0,77	0,06	0,0150	1,44	0,15	0,016
Bacillus subtilis	0,41	0,09	0,2370	1,20	0,33	0,015
Enterobacter hormaechei	0,84	0,13	0,0530	1,11	0,10	0,065
Paenibacillus peoriae	1,10	0,07	0,0060	0,91	0,10	0,031
Paenibacillus polymyxa	0,52	0,08	0,0870	0,98	0,20	0,020
Pantoae agglomerans	0,79	0,06	0,0110	1,66	0,18	0,002
Pseudomonas aeruginosa	0,86	0,07	0,0410	1,17	0,12	0,016
Pseudo frederiksbergensis	0,36	0,04	0,2970	1,04	0,19	0,012
Rhizobium sp.	0,33	0,02	0,4020	1,09	0,26	0,011
Variovorax paradoxus	0,49	0,07	0,1300	1,21	0,14	0,013

CAT	Feuille Log2(ddCt)	Feuille SE	Feuille t-st.	Racine Log2(ddCt)	Racine SE	Racine t-st.
Control	0,00	0,04	/	0,00	0,20	/
Achromobacter sp.	1,36	0,25	0,0340	0,48	0,01	0,1150
Bacillus cereus	1,46	0,23	0,0200	0,91	0,27	0,0200
Bacillus halotolerans	2,46	0,27	0,0110	0,33	0,27	0,4830
Bacillus megaterium	1,88	0,13	0,0009	0,71	0,11	0,0490
Bacillus mojavensis	1,79	0,08	0,0002	0,20	0,12	0,3480
Bacillus subtilis	1,27	0,22	0,0300	1,15	0,20	0,0090
Enterobacter hormaechei	1,15	0,06	0,0006	0,84	0,08	0,0480
Paenibacillus peoriae	1,47	0,18	0,0070	0,46	0,12	0,1120
Paenibacillus polymyxa	1,52	0,11	0,0007	0,96	0,12	0,0170
Pantoae agglomerans	2,04	0,18	0,0040	1,21	0,01	0,0070
Pseudomonas aeruginosa	2,04	0,07	0,0010	1,16	0,09	0,0110
Pseudo frederiksbergensis	1,35	0,31	0,0500	1,11	0,43	0,0420
Rhizobium sp.	1,72	0,25	0,0170	-1,29	0,35	0,0040
Variovorax paradoxus	1,54	0,37	0,0570	1,00	0,20	0,0260

SOD	Feuille Log2(ddCt)	Feuille SE	Feuille t-st.	Racine Log2(ddCt)	Racine SE	Racine t-st.
Control	0,00	0,19	/	0,00	0,11	/
Achromobacter sp.	1,61	0,42	0,0140	1,02	0,07	0,0430
Bacillus cereus	2,49	0,51	0,0040	2,41	0,36	0,0120
Bacillus halotolerans	1,78	0,31	0,0030	1,17	0,49	0,0770
Bacillus megaterium	1,59	0,36	0,0080	2,43	0,27	0,0090
Bacillus mojavensis	1,14	0,12	0,0070	0,76	0,37	0,0730
Bacillus subtilis	2,30	0,17	0,0020	1,34	0,04	0,0200
Enterobacter hormaechei	2,92	0,23	0,0008	1,96	0,37	0,0280
Paenibacillus peoriae	1,30	0,34	0,0500	1,48	0,22	0,0250
Paenibacillus polymyxa	3,95	0,21	0,0010	2,27	0,15	0,0050
Pantoae agglomerans	0,90	0,30	0,0750	2,67	0,54	0,0170
Pseudomonas aeruginosa	0,85	0,14	0,0140	0,87	0,12	0,0610
Pseudo frederiksbergensis	0,82	0,06	0,0180	1,16	0,44	0,0590
Rhizobium sp.	3,44	0,09	0,0030	1,41	0,30	0,0170
Variovorax paradoxus	2,66	0,36	0,0009	2,11	0,28	0,0080

HSP70	Feuille Log2(ddCt)	Feuille SE	Feuille t-st.	Racine Log2(ddCt)	Racine SE	Racine t-st.
Control	0,00	0,22	/	0,00	0,11	/
Achromobacter sp.	0,77	0,30	0,1080	0,73	0,07	0,1470
Bacillus cereus	1,11	0,44	0,0960	0,95	0,15	0,0040
Bacillus halotolerans	1,08	0,32	0,0580	1,10	0,11	0,0540
Bacillus megaterium	0,51	0,11	0,0330	1,16	0,31	0,0230
Bacillus mojavensis	0,59	0,13	0,0390	0,74	0,13	0,2160
Bacillus subtilis	0,73	0,03	0,0070	0,87	0,06	0,1440
Enterobacter hormaechei	0,82	0,05	0,0030	0,42	0,10	0,2450
Paenibacillus peoriae	0,98	0,24	0,0290	0,39	0,11	0,2760
Paenibacillus polymyxa	0,88	0,22	0,0310	1,12	0,14	0,0070
Pantoae agglomerans	0,80	0,12	0,0110	1,52	0,24	0,0130
Pseudomonas aeruginosa	1,48	0,02	0,0010	0,85	0,35	0,1730
Pseudo frederiksbergensis	1,38	0,31	0,0170	1,12	0,25	0,0008
Rhizobium sp.	0,79	0,05	0,0040	-3,21	0,51	0,0040
Variovorax paradoxus	0,39	0,22	0,2330	0,67	0,09	0,1240

GSL22	Feuille Log2(ddCt)	Feuille SE	Feuille t-st.	Racine Log2(ddCt)	Racine SE	Racine t-st.
Control	0,00	0,11	/	0,00	0,15	/
Achromobacter sp.	1,58	0,44	0,0830	-1,27	0,25	0,0260
Bacillus cereus	0,76	0,02	0,0360	-1,33	0,32	0,0170
Bacillus halotolerans	1,58	0,46	0,0960	-1,26	0,22	0,0200
Bacillus megaterium	0,84	0,30	0,1320	-1,24	0,24	0,0230
Bacillus mojavensis	1,41	0,19	0,0140	-1,02	0,17	0,0420
Bacillus subtilis	1,28	0,04	0,0050	-1,00	0,22	0,0430
Enterobacter hormaechei	0,89	0,13	0,0170	-1,00	0,17	0,0530
Paenibacillus peoriae	1,50	0,16	0,0060	-1,40	0,08	0,0290
Paenibacillus polymyxa	1,21	0,12	0,0060	-1,22	0,40	0,0310
Pantoae agglomerans	1,36	0,18	0,0190	-1,05	0,17	0,1290
Pseudomonas aeruginosa	1,15	0,17	0,0510	-0,81	0,21	0,0680
Pseudo frederiksbergensis	1,00	0,19	0,0330	-1,04	0,16	0,0390
Rhizobium sp.	0,56	0,43	0,3520	-2,03	0,52	0,0050
Variovorax paradoxus	0,43	0,09	0,1500	-1,29	0,39	0,0320

ACS1	Feuille Log2(ddCt)	Feuille SE	Feuille t-st.	Racine Log2(ddCt)	Racine SE	Racine t-st.
Control	0,00	0,22	/	0,00	0,26	/
Achromobacter sp.	-1,17	0,19	0,0040	-0,73	0,03	0,035
Bacillus cereus	-1,43	0,23	0,0050	-0,95	0,03	0,016
Bacillus halotolerans	-0,35	0,28	0,3360	-0,13	0,14	0,593
Bacillus megaterium	-0,70	0,09	0,0120	-0,42	0,04	0,017
Bacillus mojavensis	-0,35	0,06	0,0660	-0,92	0,10	0,071
Bacillus subtilis	-0,38	0,11	0,0720	-0,99	0,15	0,074
Enterobacter hormaechei	-1,24	0,12	0,0030	-1,12	0,29	0,080
Paenibacillus peoriae	-0,25	0,13	0,1680	-1,32	0,09	0,012
Paenibacillus polymyxa	-0,33	0,09	0,0750	-1,08	0,06	0,028
Pantoae agglomerans	-0,23	0,11	0,1850	-0,10	0,04	0,386
Pseudomonas aeruginosa	-0,17	0,11	0,4720	-0,79	0,08	0,026
Pseudo frederiksbergensis	-0,59	0,04	0,0280	-0,64	0,18	0,022
Rhizobium sp.	-0,63	0,08	0,0150	-2,45	0,38	0,046
Variovorax paradoxus	-1,74	0,36	0,0190	-0,99	0,13	0,004

PIE	Feuille Log2(ddCt)	Feuille SE	Feuille t-st.	Racine Log2(ddCt)	Racine SE	Racine t-st.
Control	0,00	0,07	/	0,00	0,12	/
Achromobacter sp.	-2,65	0,42	0,0040	-2,05	0,34	0,0620
Bacillus cereus	-1,06	0,10	0,0600	-0,94	0,30	0,0310
Bacillus halotolerans	-1,40	0,22	0,0220	-1,39	0,14	0,0340
Bacillus megaterium	-1,92	0,33	0,0110	-1,12	0,18	0,1570
Bacillus mojavensis	-1,99	0,16	0,0110	-1,26	0,10	0,0940
Bacillus subtilis	-1,19	0,28	0,0400	-0,91	0,25	0,2260
Enterobacter hormaechei	-1,27	0,24	0,0290	-1,51	0,39	0,0350
Paenibacillus peoriae	-0,73	0,09	0,1110	-1,95	0,21	0,0010
Paenibacillus polymyxa	-1,26	0,28	0,0330	-2,10	0,34	0,0020
Pantoae agglomerans	-0,69	0,24	0,1300	-0,99	0,35	0,2450
Pseudomonas aeruginosa	-0,68	0,27	0,1800	-1,31	0,24	0,0220
Pseudo frederiksbergensis	-0,69	0,19	0,1220	-0,94	0,55	0,2420
Rhizobium sp.	-0,58	0,26	0,2000	-1,24	0,19	0,0300
Variovorax paradoxus	-1,18	0,22	0,0370	-1,61	0,35	0,0020

AOS	Feuille Log2(ddCt)	Feuille SE	Feuille t-st.	Racine Log2(ddCt)	Racine SE	Racine t-st.
Control	0,00	0,19	/	0,00	0,08	/
Achromobacter sp.	0,15	0,03	0,3850	0,31	0,49	0,752
Bacillus cereus	0,73	0,21	0,0660	0,84	0,13	0,073
Bacillus halotolerans	1,57	0,19	0,0050	0,75	0,08	0,090
Bacillus megaterium	1,47	0,21	0,0090	1,73	0,41	0,015
Bacillus mojavensis	0,90	0,28	0,0720	1,70	0,32	0,016
Bacillus subtilis	1,28	0,34	0,0600	1,55	0,17	0,027
Enterobacter hormaechei	0,89	0,07	0,0110	0,83	0,10	0,012
Paenibacillus peoriae	0,73	0,21	0,0590	1,22	0,33	0,023
Paenibacillus polymyxa	0,21	0,12	0,3190	0,17	0,03	0,746
Pantoae agglomerans	1,40	0,12	0,0020	1,01	0,10	0,037
Pseudomonas aeruginosa	1,71	0,28	0,0140	0,61	0,24	0,133
Pseudo frederiksbergensis	0,98	0,10	0,0060	0,49	0,14	0,032
Rhizobium sp.	0,10	0,09	0,5920	1,99	0,34	0,003
Variovorax paradoxus	0,90	0,08	0,0090	2,28	0,47	0,016

LOX	Feuille Log2(ddCt)	Feuille SE	Feuille t-st.	Racine Log2(ddCt)	Racine SE	Racine t-st.
Control	0,00	0,28	/	0,00	0,29	/
Achromobacter sp.	1,46	0,09	0,0040	-0,58	0,12	0,418
Bacillus cereus	0,60	0,11	0,0120	-1,68	0,48	0,017
Bacillus halotolerans	1,77	0,44	0,0550	-1,33	0,21	0,064
Bacillus megaterium	1,06	0,15	0,0210	-1,68	0,40	0,032
Bacillus mojavensis	1,02	0,32	0,1030	-0,57	0,22	0,448
Bacillus subtilis	1,27	0,30	0,0660	-2,98	0,46	0,003
Enterobacter hormaechei	0,79	0,17	0,0310	-1,58	0,57	0,014
Paenibacillus peoriae	0,82	0,24	0,0840	-1,27	0,03	0,169
Paenibacillus polymyxa	0,43	0,25	0,2420	-2,23	0,43	0,174
Pantoae agglomerans	1,76	0,27	0,0170	-1,90	0,51	0,194
Pseudomonas aeruginosa	2,48	0,32	0,0130	-1,52	0,35	0,110
Pseudo frederiksbergensis	2,11	0,43	0,0320	-1,64	0,75	0,099
Rhizobium sp.	0,95	0,21	0,0630	3,22	0,59	0,003
Variovorax paradoxus	1,42	0,20	0,0230	2,60	0,30	0,007
