# Supplementary material for: Evaluation of Tunisian wheat endophytes as plant growth promoting bacteria and biological control agents against Fusarium culmorum
Source: PLoS One. 2024 May 17;19(5):e0300791. doi: 10.1371/journal.pone.0300791 (PMC11101125; doi:10.1371/journal.pone.0300791)
Supplement: S3 Fig — (PDF) [file pone.0300791.s003.pdf]

# S3 Figure.

**S3 Figure. Macroscopic assessment of the dual confrontations of each rhizobacteria against *F. culmorum*, and semi-quantification of all CDWEs activities studied for each strain.**

- Dual confrontation Rhizobacteria/*F. culmorum* in axenic condition
- Amylase activity
- Catalase activity
- Cellulase activity
- Pectinase activity
- Protease activity

# ***Dual confrontation***

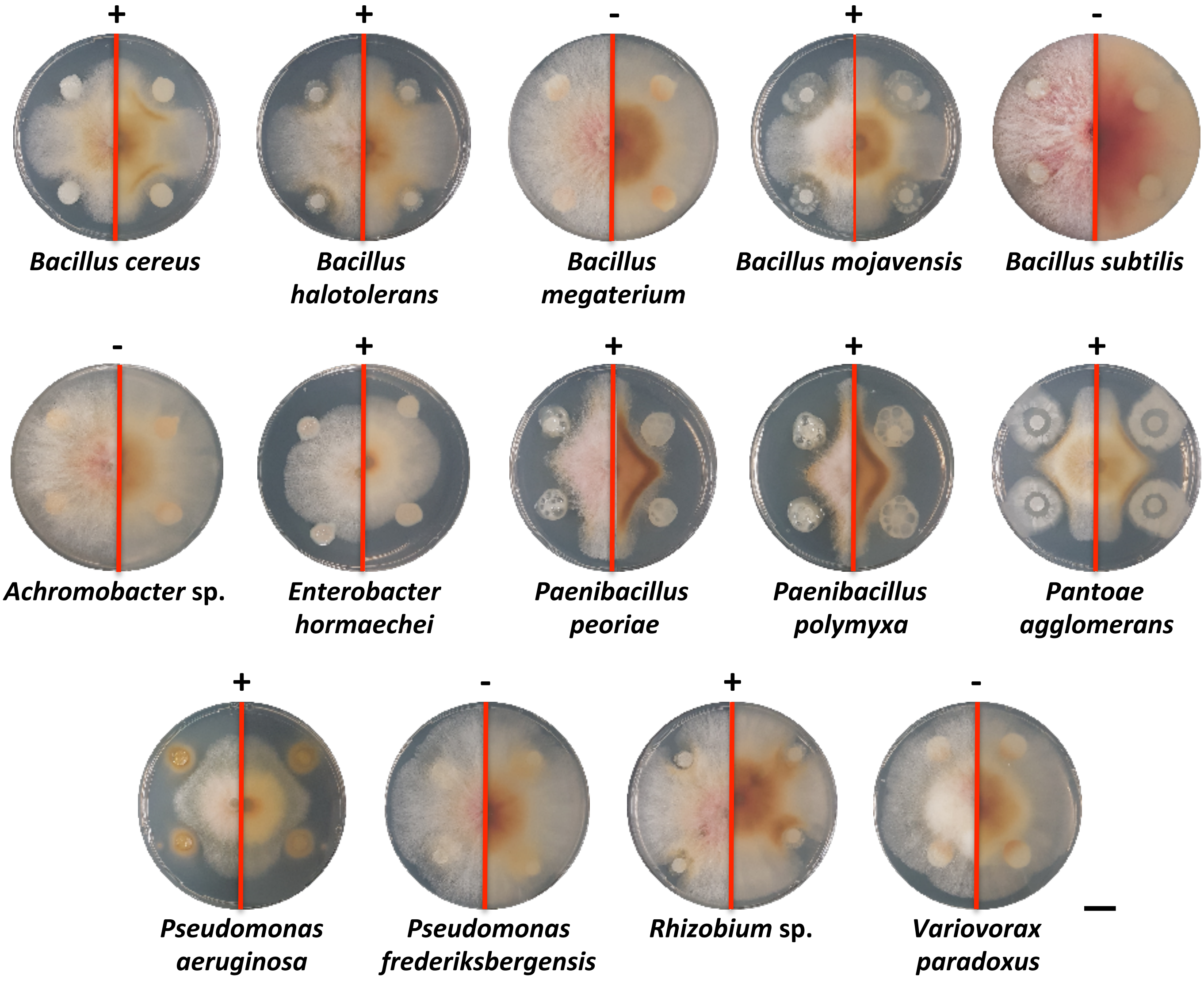


Effect of root endophytic bacterial strains on the growth kinetic of *Fusarium culmorum* in axenic culture. ("-») No inhibition; ("+") Positive inhibition. On either side of the red line, are shown the macroscopic aspects of the microorganisms that can be observed from upper (right) or back side (left) of the culture plates. Strain confrontations were recorded 5 days after microorganism development at 25°C on PDA medium. The scale bar in the last photograph represents 1 cm.

# ***Amylase activity***

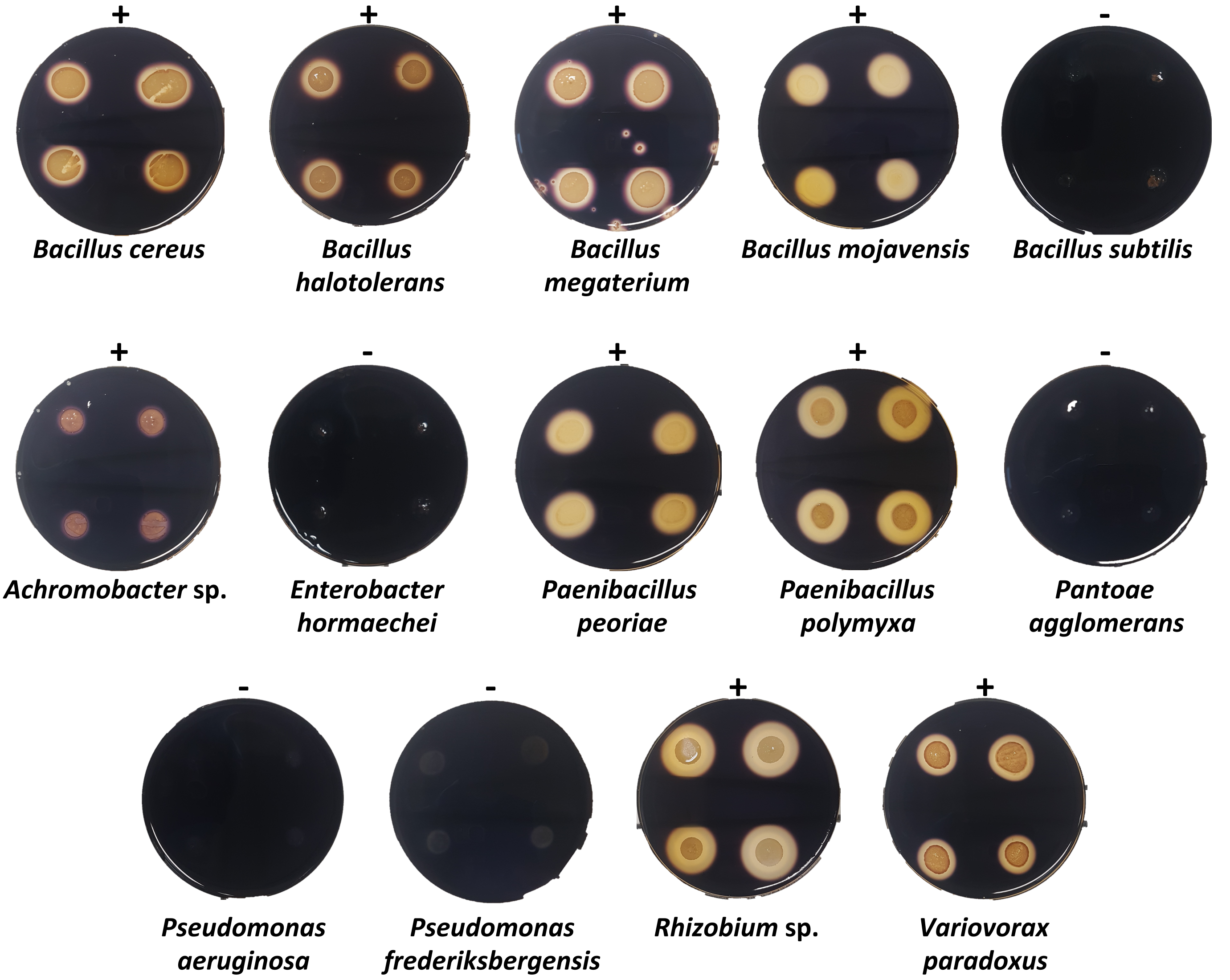


Semi-quantitative calibration of Amylase activity for each bacterial endophyte isolated on roots from the old wheat variety Florence Aurore. ("-") Negative Amylase activity; ("+") Positive Amylase activity, materialized by a translucent zone around the bacterial colonies.

# ***Catalase activity***

| *Control (water)* | *Achromobacter sp.* | *Bacillus cereus* | *Bacillus halotolerans* | *Bacillus megaterium* | *Bacillus Mojavensis* | *Bacillus subtilis* | *Enterobacter hormaechei* | *Paenibacillus peoriae* | *Paenibacillus polymyxa* | *Pantoea agglomerans* | *Pseudomonas aeruginosa* | *Pseudomonas Frederiksbergensis* | *Rhizobium sp.* | *Variovorax paradoxus* |
|---|---|---|---|---|---|---|---|---|---|---|---|---|---|---|
| ***Catalase activity*** | | | | | | | | | | | | | | |
| / | + | + | + | + | + | + | + | + | + | + | + | + | + | + |
| / | 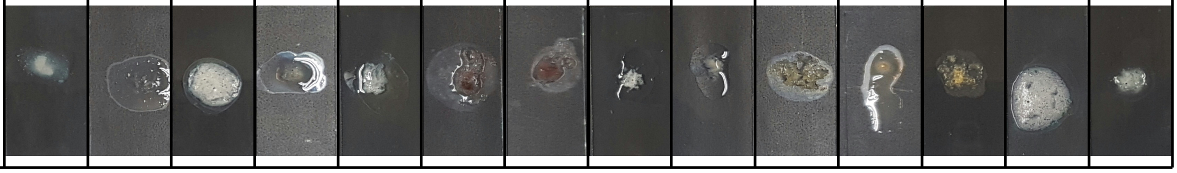 | | | | | | | | | | | | | |

Semi-quantitative calibration of Catalase activity for each bacterial endophyte isolated on roots from the old wheat variety Florence Aurore. ("-") Negative Catalase activity; ("+") Positive Catalase activity, materialized by the production of $O_2$ bubbles. The bacteria were taken from a fresh colony and placed on a petri slide in the presence of hydrogen peroxide.

*Fig S3.*

# ***Cellulase activity***

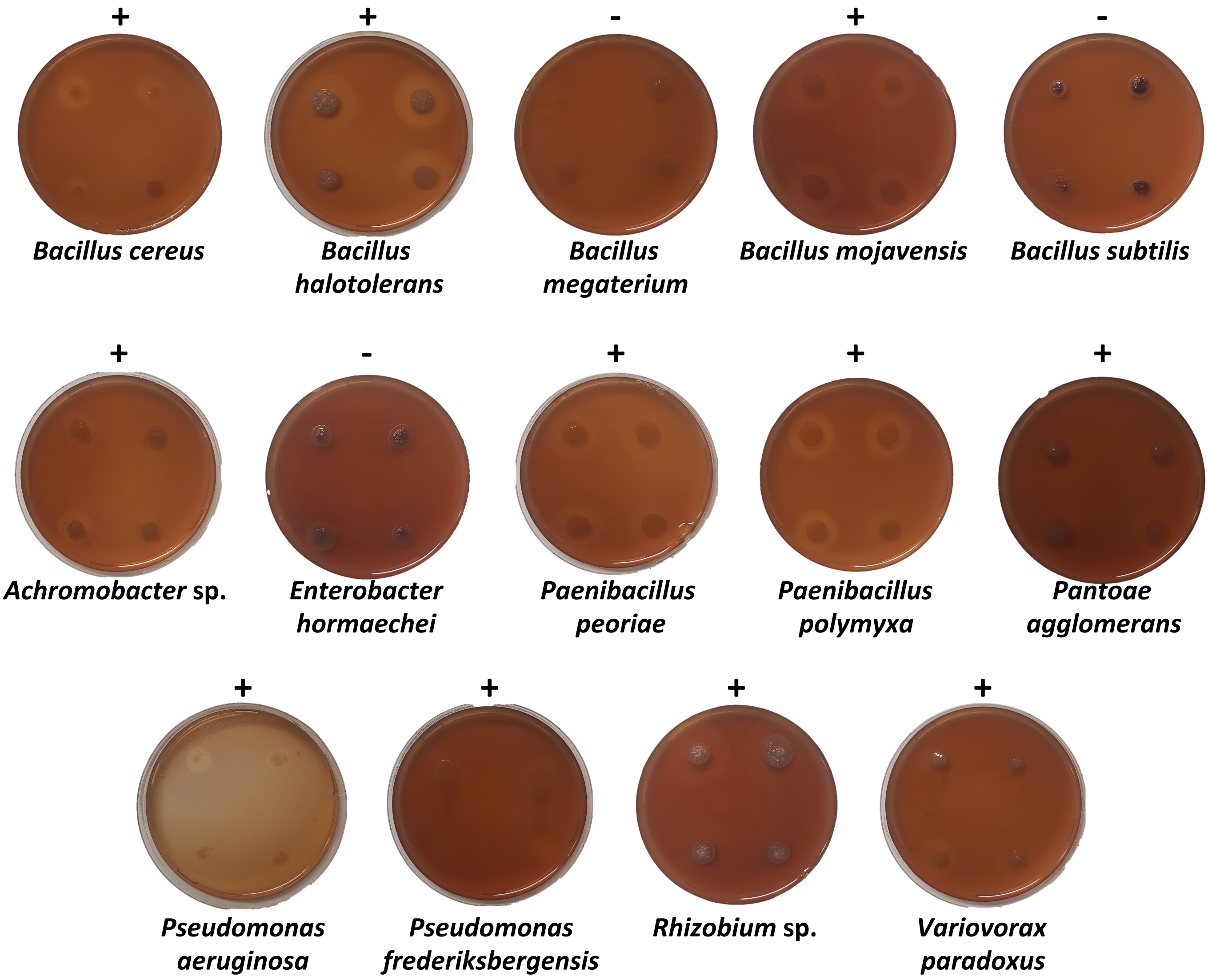


Semi-quantitative calibration of Cellulase activity for each bacterial endophyte isolated on roots from the old wheat variety Florence Aurore. ("-") Negative Cellulase activity, ("+") Positive Cellulase activity, materialized by a translucent zone around the bacterial colonies.

# ***Pectinase activity***

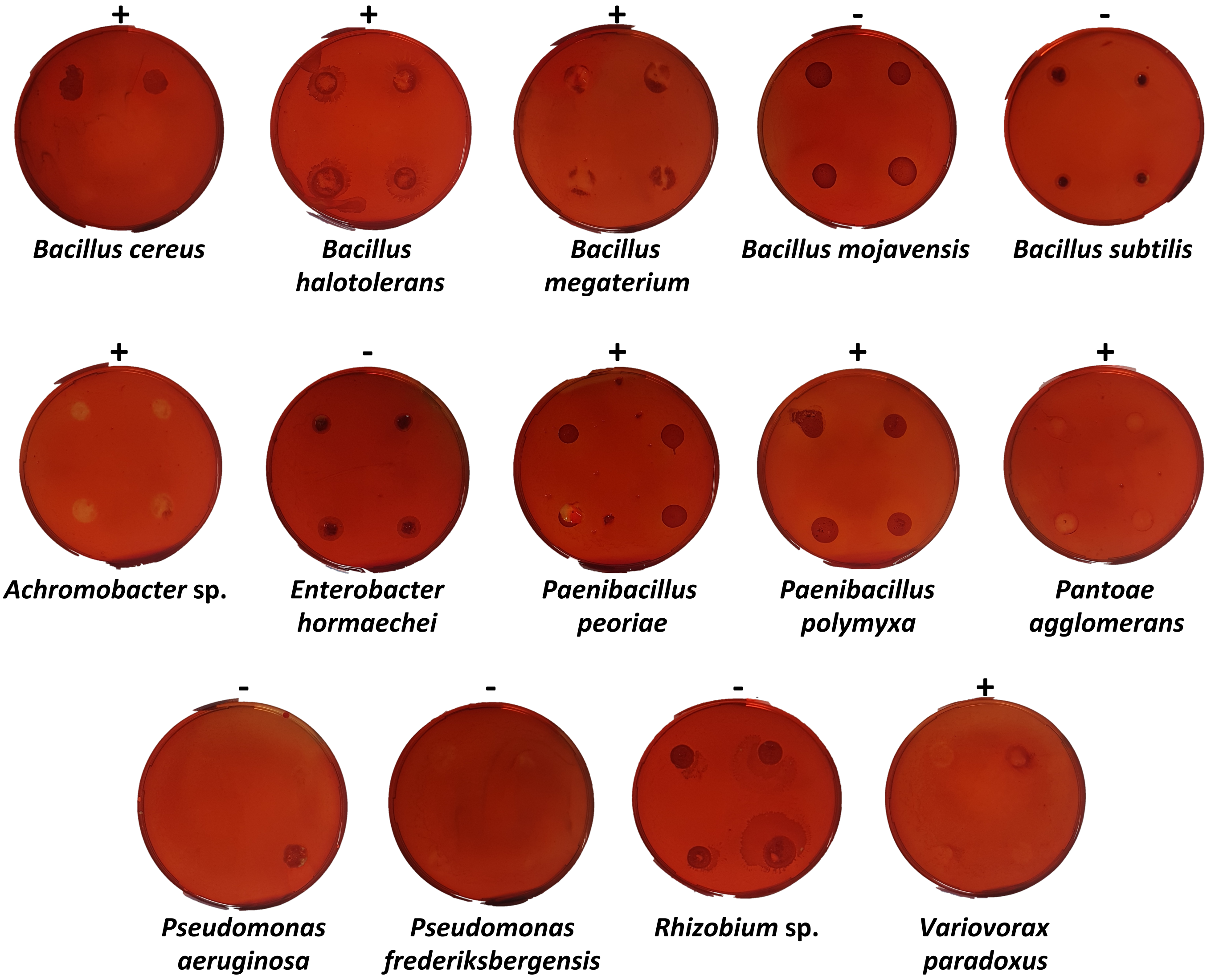


Semi-quantitative calibration of Pectinase activity for each bacterial endophyte isolated on roots from the old wheat variety Florence Aurore. ("-") Negative Pectinase activity; ("+") Positive Pectinase activity, materialized by a translucent zone around the bacterial colonies.

# ***Protease activity***

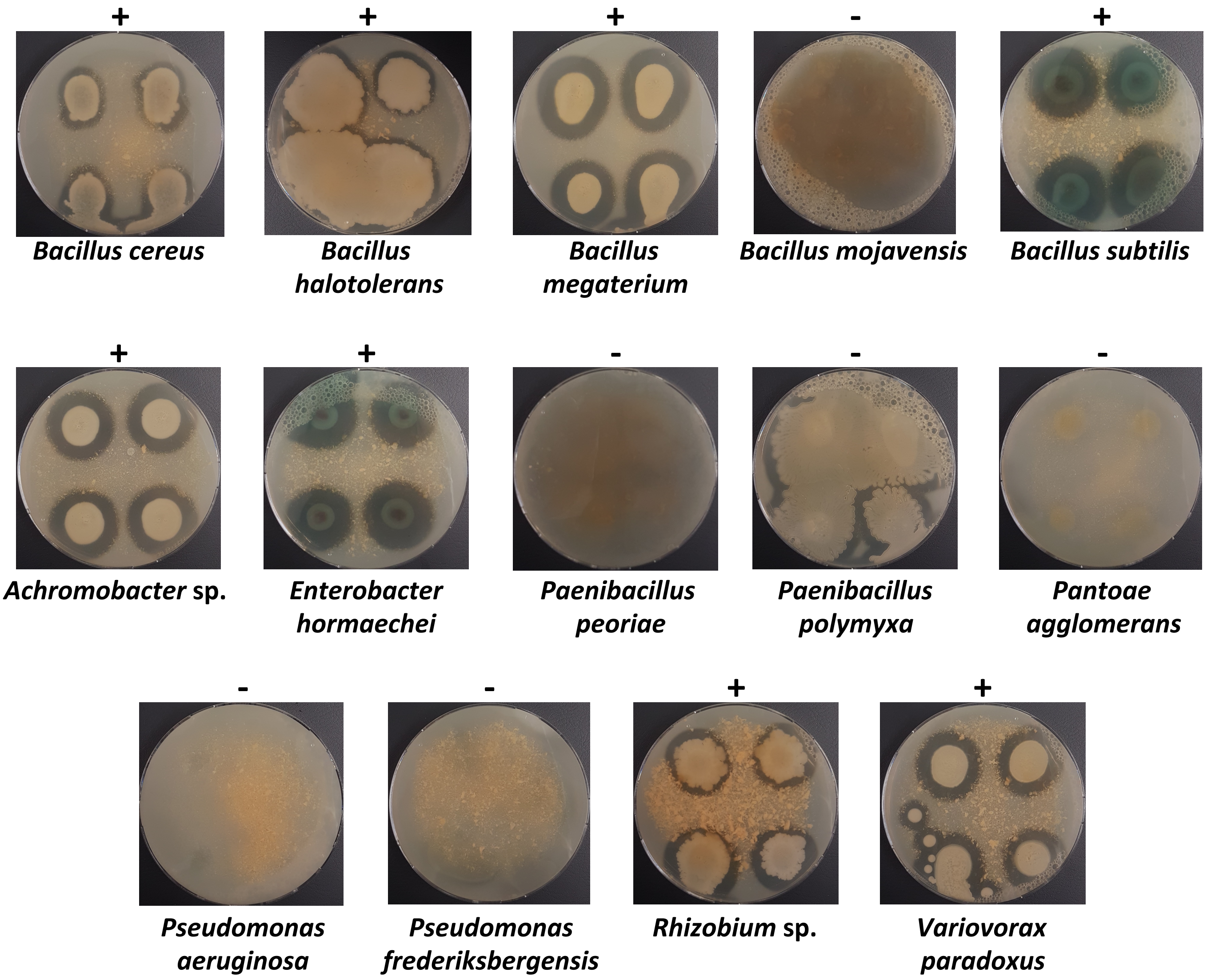


Semi-quantitative calibration of Protease activity for each bacterial endophyte isolated on roots from the old wheat variety Florence Aurore. ("-") Negative Protease activity; ("+") Positive Protease activity, materialized by a translucent zone around the bacterial colonies.
