# Supplementary material for: Evaluation of Tunisian wheat endophytes as plant growth promoting bacteria and biological control agents against Fusarium culmorum
Source: PLoS One. 2024 May 17;19(5):e0300791. doi: 10.1371/journal.pone.0300791 (PMC11101125; doi:10.1371/journal.pone.0300791)
Supplement: S1 Table — (PDF) [file pone.0300791.s004.pdf]

S1 Table. Primers used for the qPCR amplifications

Housekeeping genes (5'-3')			
**Gene, full name**	**Acronym**	**Primer (5'-3')**	**PCR efficiency**
Actine	ACT FOR	AGTGGAGGTTCTACCATGTTTCCT	2.52
	ACT REV	CACTGTATTTCCTTTCAGGTGGTG	
Glyceraldehyde-3-Phosphate Dehydrogenase	GAPDH FOR	AAGGGCATTTTGGGTTACGTT	2.01
	GAPDH REV	CCTGTTGTCACCCTGGAAGTC	
RNaseL inhibitor-like	RLI FOR	TTGAGCAACTCATGGACCAG	2.09
	RLI REV	GCTTTCCAAGGCACAAACAT	
Heterogeneous nuclear ribonucleoprotein	RNP FOR	TCACCTTCGCCAAGCTCAGAACTA	2.05
	RNP REV	AGTTGAACTTGCCCGAAAC	
26S ribosomal RNA	26S FOR	GCTGGCTCGTTCAACTGATG	2.15
	26S REV	GGACCAAGCGTTCTGATTACTC	

Target genes (5'-3')			
**Gene, full name**	**Acronym**	**Primer (5'-3')**	**PCR efficiency**
Allene oxide synthase	AOS FOR	AGGCCGGAGAGAAGTTCCAC	1.92
	AOS REV	CCGACTTGGTCAGCTCCATC	
Aminocyclopropane (ACC) synthase	ACS1 FOR	GAGGTTGTGAACGAGCTAGAAG	1.89
	ACS1 REV	CTCGAAACCCTGCAAGAGATAG	
Aquaporin (PIP-like)	AQP FOR	TGGTCAGACCACTGGATCTTC	2.07
	AQP REV	TGGCATCTTCTTTGCAGCAG	
Catalase	CAT FOR	CACCTGGTGGAGAAGATCGC	1.96
	CAT REV	TCACCTCGAAGAAGCCCTTG	
Chitinase (PR-4)	CHT FOR	GGGTGGACCTGCTGAACAAT	2.02
	CHT REV	AGAACCATATCGCCGTCTTGA	
Cytokinin oxidases/dehydrogenase	CKX FOR	GGAGGTGGCGCTGGACAAGATC	1.91
	CKX REV	GCAGAACCGCAGTATCTTCTGGT	
Defensin (PR-12)	DEF FOR	ATGTCCGTGCCTTTTGCTA	1.93
	DEF REV	CCAAACTACCGAGTCCCCG	
Isopentenyltransferase 5	IPT5 FOR	CGAACTAGCTGTCATGCCATAG	2.07
	IPT5 REV	AGAGCTTGACCGTAGCAGTA	
Enhanced Disease Susceptibility 1	EDS1 FOR	TACATGAGGCACCTGCCGCAGT	2.01
	EDS1 REV	GGTTCAACTCCAGGGCTTCGTTG	
glucan synthase-like 22 (callose synthase)	GSL22 FOR	GTCTGGGGGCGATTTTA	2.18
	GSL22 REV	GCTGTATGACGAACTGCTGA	
Heat Shock Protein 70-like (HSP-70)	HSP70 FOR	CGACTCGGAGCGTCTCATC	1.72
	HSP REV	TGTCACTCTGACAGGGCATCA	
Lipid transfer protein (PR-14)	LTP FOR	ACGTAGGTACTCCTCTCGCTGT	2.19
	LTP REV	GTTGATCGACCACTTCTTCTCA	
Lipoxygenase	LOX FOR	GGGCACCAAGGAGTACAAGGA	2.18
	LOX REV	GCTCGTGATGGTGTGGATGA	
Receptor Like Kinase (LRR-RLK class)	RLK FOR	TGCTGCATTCCTGATCAAGG	2.01
	RLK REV	GGTGAAATTTGGTGTTGCAC	
Mitogen-activated protein kinase 3	MAPK3 FOR	TACATGAGGCACCTGCCGCAGT	2.32
	MAPK3 REV	GGTTCAACTCCAGGGCTTCGTTG	
Non Expresser of Pathogenesis Related 1	NPR1 FOR	GCTTGTCAGGATGCTGCTC	1.78
	NPR1 REV	GAACAGTATAACCTCTTGGGTTTC	
Phenylalanine ammonia lyase	PAL FOR	GGCCCACAAATCGAAGTTATTC	2.09
	PAL REV	GGAAGTTGCCACCGTGGAT	
Polyphenol oxydase	PPO FOR	GTCCGGACGAACAAGAAGAA	2.09
	PPO REV	GGCGATGCTTCACTTGGTAT	
Peroxydase (PR-9)	POX FOR	TGCTTTGTCCAAGGCTGTGA	1.97
	POX REV	GACCCGCGTTTTGTTCCA	
Pathogenesis related Protein 1 (PR-1)	PR-1 FOR	GAATGCAGACGCCCAAGCTA	1.78
	PR-1 REV	GCACGGGCAGCGTTGT	
Pathogenesis related Protein 5 (PR-5)	PR5 FOR	CGTTGATGCTCGGGGCGCCG	1.87
	PR5 REV	AATTCGCCTCAAGACCTCAC	
Pathogenesis related Protein 17 (PR-17)	PR-17 FOR	CATCTGGACCACCTTCAACCA	1.97
	PR-17 REV	ACTGGGCTCGGAGTTGGATA	
Superoxide dismutase	SOD FOR	CGATAGCCAGATTCCTTTGACT	2.11
	SOD REV	GAAACCAGCGACCTACAACG	
Sesquiterpene synthase	SqTS FOR	GGAAAGACATGGTGCGACA	1.82
	SqTS REV	GATCTCGTGCGAGGAAGTG	
sulphur-rich Thionin-like (PR-13)	THIO FOR	TGCATCGACAAGACCGAAAA	1,78
	THIO REV	ACGCATTCGACAAAGCACTTC	
Wall-associated Kinase 6	WAK6 FOR	AGAACCTCTCGTCGCACTTC	2.14
	WAK6 REV	CCTTAGCCTGCCAAGCTCTT	
