# Supplementary material for: Evaluation of Tunisian wheat endophytes as plant growth promoting bacteria and biological control agents against Fusarium culmorum
Source: PLoS One. 2024 May 17;19(5):e0300791. doi: 10.1371/journal.pone.0300791 (PMC11101125; doi:10.1371/journal.pone.0300791)
Supplement: S2 Fig — (PDF) [file pone.0300791.s002.pdf]

S2 Figure

S2 Figure. Macroscopic assessment and quantification of all biochemical activities studied for each strain.

- Phosphate solubilization
- Zinc solubilization
- Indoles-related Production
- Ammonia Production
- HCN production

Phosphate solubilization

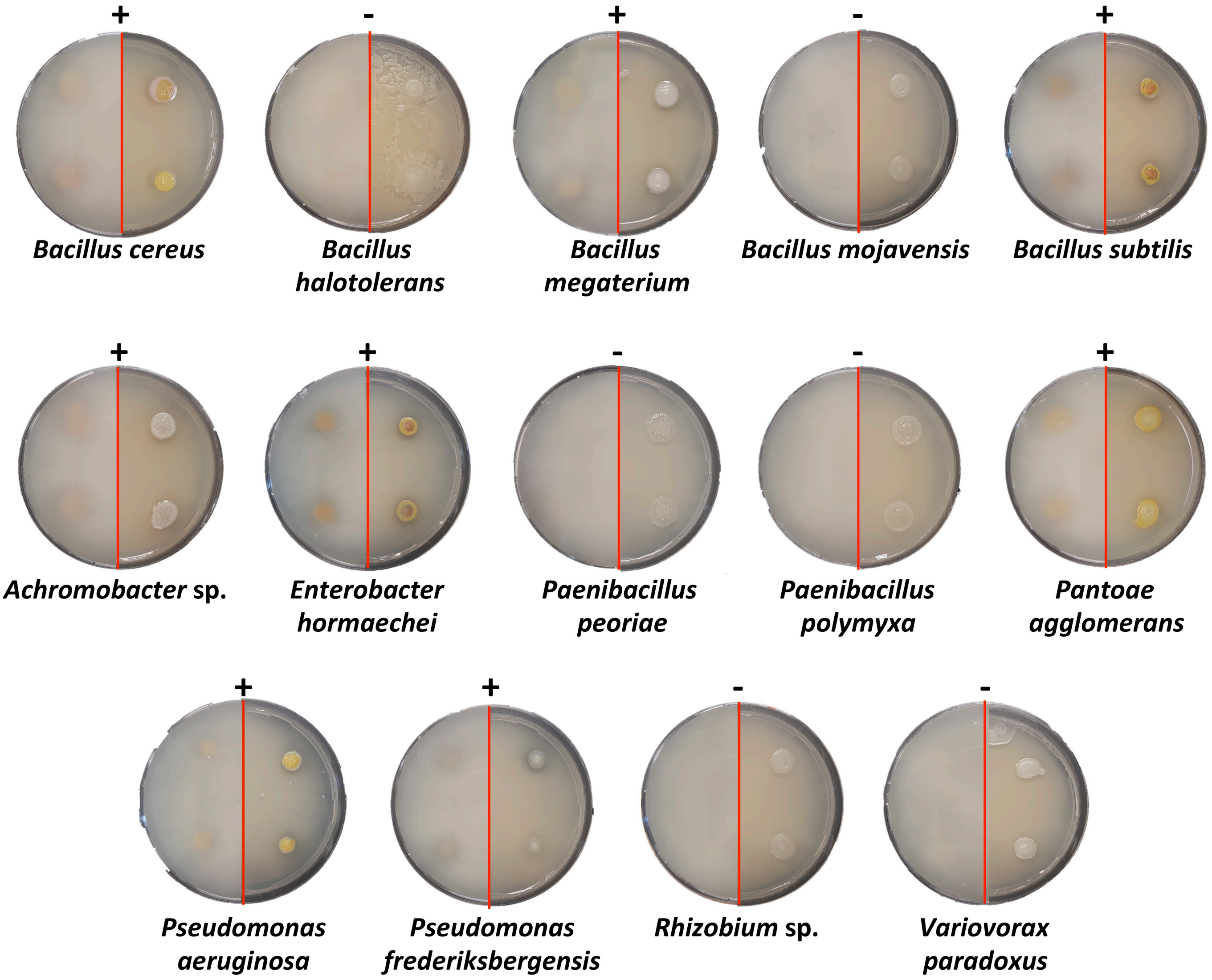

Semi-quantitative calibration of phosphate solubilization by each bacterial endophyte isolated on roots from the old wheat variety Florence Aurore. Each culture is represented by a view from below the box (left part of the red line) and from above the box (left part of the red line). ("-") negative P solubilization; ("+") Positive P solubilization, materialized by a translucent zone around the bacterial colonies.

<u>*Zinc solubilization*</u>

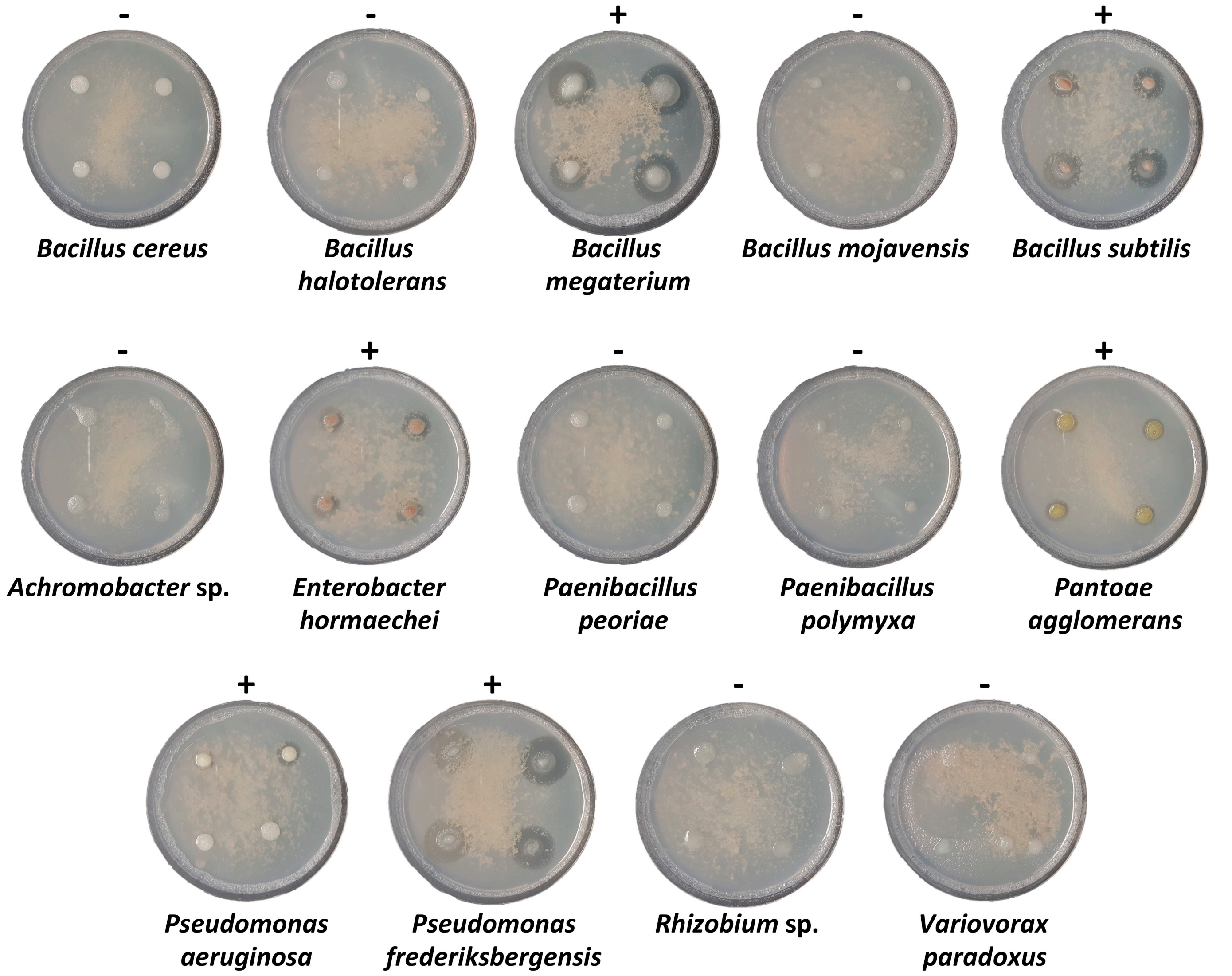

Semi-quantitative calibration of Zinc solubilization by each bacterial endophyte isolated on roots from the old wheat variety Florence Aurore. ("-") Negative Zn solubilization; ("+") Positive Zn solubilization, materialized by a translucent zone around the bacterial colonies.

Indole-related compounds and ammonia productions

	Control (water)	*Achromobacter sp.*	*Bacillus cereus*	*Bacillus halotolerans*	*Bacillus megaterium*	*Bacillus Mojavensis*	*Bacillus subtilis*	*Enterobacter hormaechei*	*Paenibacillus peoriae*	*Paenibacillus polymyxa*	*Pantoea agglomerans*	*Pseudomonas aeruginosa*	*Pseudomonas Frederiksbergensis*	*Rhizobium sp.*	*Variovorax paradoxus*
Indole-related compounds production	/	+	+	+	+	+	+	+	+	+	+	+	+	+	+
Ammonia production	/	+	+	+	+	+	+	+	+	+	+	+	+	+	+

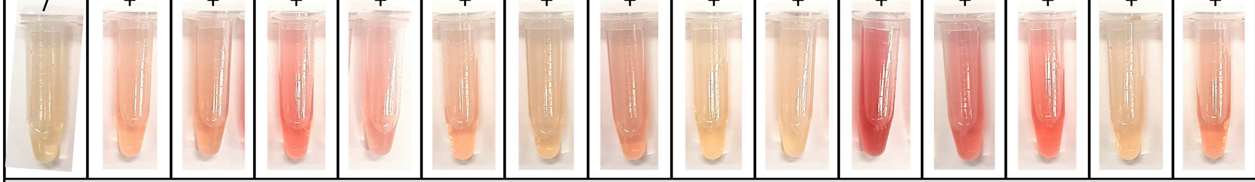

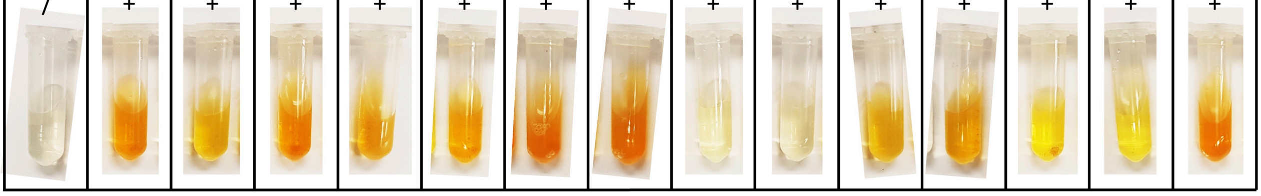

HCN production

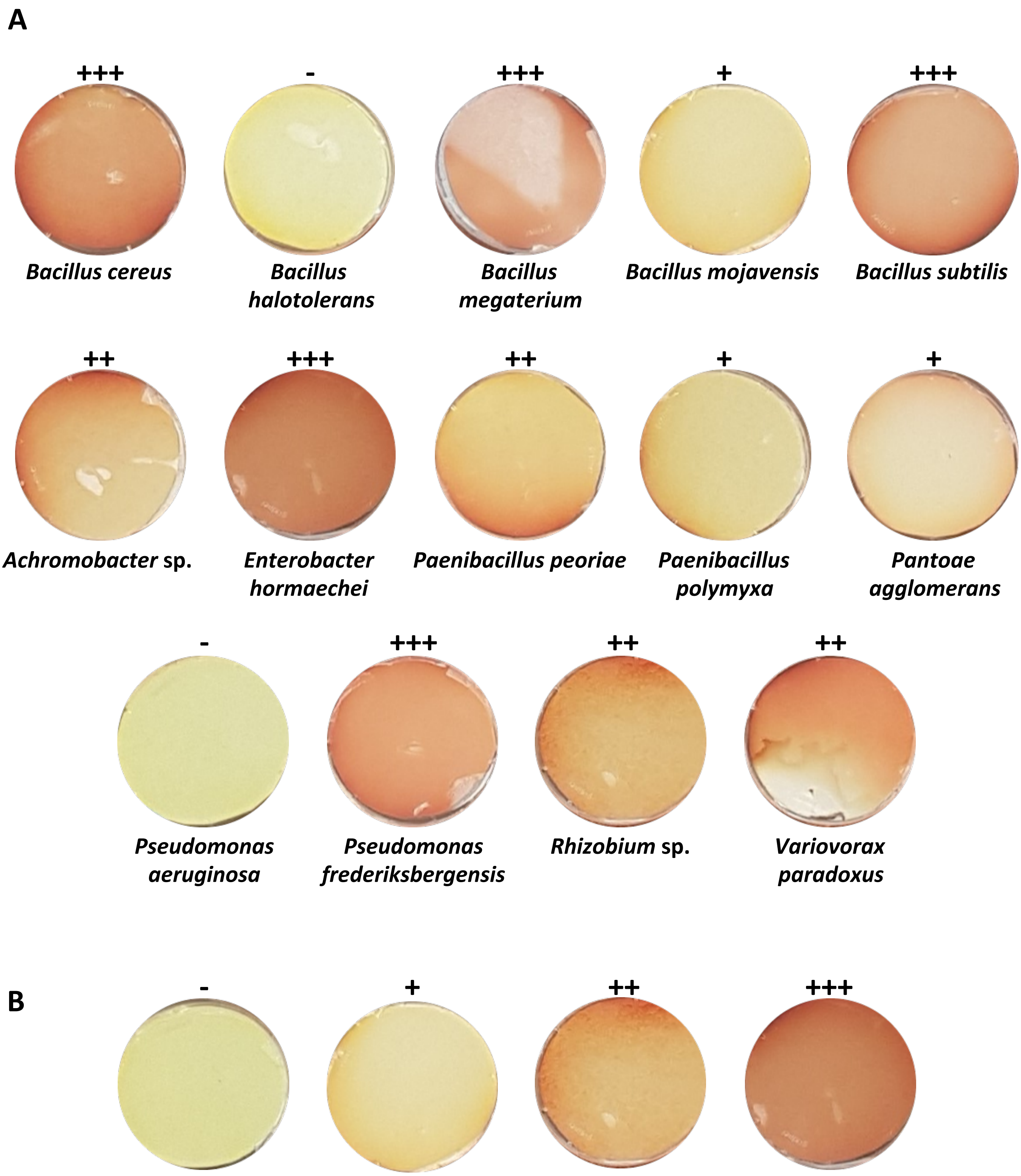

Biochemical capacities (Hydrogen cyanid, HCN) of the bacterial endophytes isolated on roots from the old wheat variety Florence Aurore. (A) HCN production by each endophyte. (**B**) Semi-quantitative calibration of HCN production ("-", negative production ; "+/++/+++", gradual production of HCN).
