# Supplementary material for: Evaluation of Tunisian wheat endophytes as plant growth promoting bacteria and biological control agents against Fusarium culmorum
Source: PLoS One. 2024 May 17;19(5):e0300791. doi: 10.1371/journal.pone.0300791 (PMC11101125; doi:10.1371/journal.pone.0300791)
Supplement: Attachment — Submitted filename: Response to Reviewers.docx [file pone.0300791.s006.docx]

2024, February 15^th^

Dear Editor and Reviewers,

Thank you for your encouraging remarks on our work.

Please find below our answers for each Reviewers comments (directly on the “View Letter” file by efficiency). All our replies are in red, as within the main text of the draft.

We hope that, to a very large extent, we lived up to your expectations. Please do not hesitate to contact us for any additional information you may require.

Thank you for your comments and your help to improve our article.

Sincerely yours,

**View Letter**

Bas du formulaire

| Date: | Jan 08 2024 09:17AM |
| --- | --- |
| To: | "Jean-Stéphane Venisse" j-stephane.venisse@uca.fr |
| From: | "PLOS ONE" plosone@plos.org |
| Subject: | PLOS ONE Decision: Revision required [PONE-D-23-35387] |

PONE-D-23-35387

Evaluation of tunisian wheat endophytes as plant growth promoting bacteria and biological control agents against Fusarium culmorum

PLOS ONE

Dear Dr. Venisse,

Thank you for submitting your manuscript to PLOS ONE. After careful consideration, we feel that it has merit but does not fully meet PLOS ONE&rsquo;s publication criteria as it currently stands. Therefore, we invite you to submit a revised version of the manuscript that addresses the points raised during the review process.

Please submit your revised manuscript by Feb 22 2024 11:59PM. If you will need more time than this to complete your revisions, please reply to this message or contact the journal office at [plosone@plos.org](mailto:plosone@plos.org). When you&#39;re ready to submit your revision, log on to https://www.editorialmanager.com/pone/ and select the &#39;Submissions Needing Revision&#39; folder to locate your manuscript file.

Please include the following items when submitting your revised manuscript:

- A rebuttal letter that responds to each point raised by the academic editor and reviewer(s). You should upload this letter as a separate file labeled &#39;Response to Reviewers&#39;.
- A marked-up copy of your manuscript that highlights changes made to the original version. You should upload this as a separate file labeled &#39;Revised Manuscript with Track Changes&#39;.
- An unmarked version of your revised paper without tracked changes. You should upload this as a separate file labeled &#39;Manuscript&#39;.

If you would like to make changes to your financial disclosure, please include your updated statement in your cover letter. Guidelines for resubmitting your figure files are available below the reviewer comments at the end of this letter.

If applicable, we recommend that you deposit your laboratory protocols in protocols.io to enhance the reproducibility of your results. Protocols.io assigns your protocol its own identifier (DOI) so that it can be cited independently in the future. For instructions see: <https://journals.plos.org/plosone/s/submission-guidelines#loc-laboratory-protocols>. Additionally, PLOS ONE offers an option for publishing peer-reviewed Lab Protocol articles, which describe protocols hosted on protocols.io. Read more information on sharing protocols at [https://plos.org/protocols?utm_medium=editorial-email&amp;utm_source=authorletters&amp;utm_campaign=protocols](https://plos.org/protocols?utm_medium=editorial-email&utm_source=authorletters&utm_campaign=protocols).

We look forward to receiving your revised manuscript.

Kind regards,

Estibaliz Sansinenea
Academic Editor
PLOS ONE

Journal requirements:

When submitting your revision, we need you to address these additional requirements.

1. Please ensure that your manuscript meets PLOS ONE&#39;s style requirements, including those for file naming. The PLOS ONE style templates can be found at

https://journals.plos.org/plosone/s/file?id=wjVg/PLOSOne_formatting_sample_main_body.pdf and

https://journals.plos.org/plosone/s/file?id=ba62/PLOSOne_formatting_sample_title_authors_affiliations.pdf

[Authors] The full article was double-checked and we made every effort to ensure that our manuscript met PLOS ONE's stylistic requirements.

2. Thank you for stating the following financial disclosure:

« This work was awarded by the I-SITE CAP 20-25 (ANR grant 16-IDEX-0001) Emergence 2017 from the University of Clermont-Auvergne, and the project "Pack Ambition International 2019" [ANR grant n° P010O003] co-financed by the University of Clermont-Auvergne and the French Region of Auvergne-Rhône Alpes »

Please state what role the funders took in the study.  If the funders had no role, please state: &quot;The funders had no role in study design, data collection and analysis, decision to publish, or preparation of the manuscript.&quot;

If this statement is not correct you must amend it as needed.

Please include this amended Role of Funder statement in your cover letter; we will change the online submission form on your behalf.

[Authors] Indeed, we confirm that "*The funders had no role in study design, data collection and analysis, decision to publish, or preparation of the manuscript*." This mention was added in the “Funding” item in the article.

3. We note that you have included the phrase &ldquo;data not shown&rdquo; in your manuscript. Unfortunately, this does not meet our data sharing requirements. PLOS does not permit references to inaccessible data. We require that authors provide all relevant data within the paper, Supporting Information files, or in an acceptable, public repository. Please add a citation to support this phrase or upload the data that corresponds with these findings to a stable repository (such as Figshare or Dryad) and provide and URLs, DOIs, or accession numbers that may be used to access these data. Or, if the data are not a core part of the research being presented in your study, we ask that you remove the phrase that refers to these data.

[Authors] We have removed the sentence from the discussion.

Additional Editor Comments:

The reviewers have commmented about this MS. One of them has suggested minor revision however the second has rejected the MS due to is lacking of novelty. I have read the MS and although the topic has been widely stidied this Ms is experimentally sound and is well written. Therefore I invite the authors to do all revisio s suggested by the reviewers answering in the best way the second referee.


[Note: HTML markup is below. Please do not edit.]

Reviewers&#39; comments:

Reviewer&#39;s Responses to Questions

**Comments to the Author**

1. Is the manuscript technically sound, and do the data support the conclusions?

The manuscript must describe a technically sound piece of scientific research with data that supports the conclusions. Experiments must have been conducted rigorously, with appropriate controls, replication, and sample sizes. The conclusions must be drawn appropriately based on the data presented.

Reviewer #1: Yes

Reviewer #2: Partly

2. Has the statistical analysis been performed appropriately and rigorously?

Reviewer #1: Yes

Reviewer #2: Yes

3. Have the authors made all data underlying the findings in their manuscript fully available?

The [PLOS Data policy](http://www.plosone.org/static/policies.action#sharing) requires authors to make all data underlying the findings described in their manuscript fully available without restriction, with rare exception (please refer to the Data Availability Statement in the manuscript PDF file). The data should be provided as part of the manuscript or its supporting information, or deposited to a public repository. For example, in addition to summary statistics, the data points behind means, medians and variance measures should be available. If there are restrictions on publicly sharing data&mdash;e.g. participant privacy or use of data from a third party&mdash;those must be specified.

Reviewer #1: Yes

Reviewer #2: Yes

4. Is the manuscript presented in an intelligible fashion and written in standard English?

PLOS ONE does not copyedit accepted manuscripts, so the language in submitted articles must be clear, correct, and unambiguous. Any typographical or grammatical errors should be corrected at revision, so please note any specific errors here.

Reviewer #1: Yes

Reviewer #2: Yes

5. Review Comments to the Author

Please use the space provided to explain your answers to the questions above. You may also include additional comments for the author, including concerns about dual publication, research ethics, or publication ethics. (Please upload your review as an attachment if it exceeds 20,000 characters)

**Reviewer #1:** Dear Corresponding Author
I checked your paper. It is well-written and in my idea everything is going excellent. I have some minor comments to improve your paper more:
1) In Figure 4 part B please use heat-map instead of +/- table.

[Authors] Figure 4 has been revised to make it easier to read, in particular by adding a color code to the "+" and "-" symbols. This change is similar to a heatmap, as rightly suggested by reviewer 1.

2) About statistical analysis are you sure your data had normal distribution? We will use ANOVA just for normal data.

[Authors] A normality test was carried out for each series of values. Concerning ANOVA, normality of the data (residuals) was assessed by both visual inspection (Q-Q plot : quantile- quantile plot plot) and significance test (Shapiro-Wilk's test). In most cases, normality of the residuals was respected. In case the residuals were not normal, Tukey's Ladder of power transformation was used. This approach uses a simple power transformation on the data set (square root transformation, cube root transformation, etc.). Another thing, homogeneity (homoscedasticity) of variances test across groups also has to be carried out (Levene's test). In this respect, we have added a clarification in the statistical paragraph of the MM (lines 357-359).

**Reviewer #2:** The research paper titled &ldquo;Evaluation of tunisian wheat endophytes as plant growth promoting bacteria and biological control agents against Fusarium culmorum&rdquo; was thoroughly examined.

Regrettably, it represents a competent yet repetitive investigation of endophytes as PGPR agents.

Numerous similar articles are readily accessible in databases such as Google Scholar, so the rationale behind the authors&rsquo; decision to replicate these types of experiments is not readily comprehensible to me.

It is inherently apparent from the outset that endophytes exhibit favorable effects on pathogenic agents.

[Authors] Thank you for emphasizing the rigor and the competence of our study. We are aware of the repetitive aspect of our study at first sight. However, it’s important to note that each pathosystem (as in this case Wheat - *F. culmorum*) possesses its own intrinsic specificity. Furthermore, the isolated strains exhibit their own singularity, which, in most cases, can be different between strains belonging to the same phyla. This variability is particularly pronounced when they evolve in a tripartite pathological context, which remains relatively complex.

You are right to point out that many similar articles are easily accessible in different databases. However, we believe it’s relevant to focus on isolating, testing and publishing the PGP and BCA capacities of the new bacterial strains isolated within a specific biotope. This approach is justified by the diverse effects that different PGPR strains can have on plants and their ability to protect against specific pathogens.

Furthermore, it's important to mention that this work constitutes a chapter in M. SAADAOUI's thesis. As such, this article represents a significant aspect of his academic development, and we are immensely proud of the positive evaluation it has received. We sincerely appreciate your recognition and encouragement.

6. PLOS authors have the option to publish the peer review history of their article ([what does this mean?](https://journals.plos.org/plosone/s/editorial-and-peer-review-process#loc-peer-review-history)). If published, this will include your full peer review and any attached files.


If you choose &ldquo;no&rdquo;, your identity will remain anonymous but your review may still be made public.


**Do you want your identity to be public for this peer review?** For information about this choice, including consent withdrawal, please see our [Privacy Policy](https://www.plos.org/privacy-policy).

Reviewer #1: No

Reviewer #2: **Yes:** Reza Khakvar

[NOTE: If reviewer comments were submitted as an attachment file, they will be attached to this email and accessible via the submission site. Please log into your account, locate the manuscript record, and check for the action link &quot;View Attachments&quot;. If this link does not appear, there are no attachment files.]

While revising your submission, please upload your figure files to the Preflight Analysis and Conversion Engine (PACE) digital diagnostic tool, <https://pacev2.apexcovantage.com/>. PACE helps ensure that figures meet PLOS requirements. To use PACE, you must first register as a user. Registration is free. Then, login and navigate to the UPLOAD tab, where you will find detailed instructions on how to use the tool. If you encounter any issues or have any questions when using PACE, please email PLOS at [figures@plos.org](mailto:figures@plos.org). Please note that Supporting Information files do not need this step.

<span style="font-style: italic; font-size: 90%;">In compliance with data protection regulations, you may request that we remove your personal registration details at any time. [(Remove my information/details)](https://www.editorialmanager.com/pone/login.asp?a=r). Please contact the publication office if you have any questions.

Haut du formulaire

Bas du formulaire
